# Engineering megabase-sized genomic deletions with MACHETE (Molecular Alteration of Chromosomes with Engineered Tandem Elements)

**DOI:** 10.1038/s41596-024-00953-9

**Published:** 2024-02-07

**Authors:** Francisco M. Barriga, Scott W. Lowe

**Affiliations:** 1: Systems Oncology Program, Vall D´Hebron Institute of Oncology (VHIO), Vall D´Hebron Hospital Campus, Barcelona, Spain.; 2: Cancer Biology & Genetics Program, Sloan Kettering Institute, Memorial Sloan Kettering Cancer Center, New York, NY, USA.; 3: Howard Hughes Medical Institute, Chevy Chase, MD, USA

## Abstract

Elimination of large genomic regions has been enabled by the advent of site-specific nucleases. However, as the intended deletions get larger, the efficiency of successful engineering decreases to a point where it is not feasible to retrieve edited cells due to the rarity of on-target events. To address this issue, we developed Molecular Alteration of Chromosomes with Engineered Tandem Elements (MACHETE). MACHETE is a CRISPR-Cas9 based system involving two stages: the initial insertion of a bicistronic positive/negative selection cassette to the locus of interest. This is followed by the introduction of single guide RNAs (sgRNAs) flanking the knock in cassette to engineer the intended deletion, where only cells that have lost the locus survive the negative selection. In contrast to other approaches optimizing the activity of sequence-specific nucleases, MACHETE selects for the deletion event itself, thus greatly enriching for cells with the engineered alteration. The procedure routinely takes 4-6 weeks from design to selection of polyclonal populations bearing the deletion of interest. We have successfully deployed MACHETE to engineer deletions of up to 45 Mb, as well as the rapid creation of allelic series to map the relevant activities within a locus. This protocol details the design and step-by-step procedure to engineer megabasesized deletions in cells of interest, with potential application for cancer genetics, transcriptional regulation, genome architecture, and beyond.

## Editorial Summary

Here the authors introduce MACHETE (Molecular Alteration of Chromosomes with Engineered Tandem Elements), a CRISPR-Cas9 based system for the efficient deletion of megabase-sized genomic regions.

## Tweet

Engineering megabase-sized genomic deletions with MACHETE guides the implementation of the method developed by @barrigafm @LoweLabMSKCC

## Cover Teaser

Engineering large genomic deletions with MACHETE

## INTRODUCTION

Genetic approaches have been fundamental to dissect the contribution of specific regions of the genome to biological phenotypes. Indeed, the development of methods to disrupt specific sequences have been instrumental to understand the role of distinct loci in development, physiology, and disease. Among genetic approaches, zinc finger nucleases (ZFNs)^1^, transcription activator-like effector nucleases (TALENs)^2^, and Clustered Regularly Interspaced Short Palindromic Repeats directed Cas9 (CRISPR-Cas9)^3^ have been used for sequence-specific manipulation of the genome across multiple model organisms and cellular systems^4,5,6,7,8,9^. The most frequent use of nucleases has been the introduction of double strand breaks (DSBs), which disrupt the target DNA sequence by engaging error-prone non homologous end joining (NHEJ). Among these technologies, the ease of design and implementation of CRISPR-Cas9 has enabled knockouts of individual genes to be created on a genome wide scale across models and experimental settings ^7,10-12^.

Despite the many advances in understanding the contribution of individual genes to cellular phenotypes, dissecting the function of larger regions of DNA has remained a challenge. This is due to the higher complexity of eliminating regions of the genome when comparing with sequence disruptions through NHEJ. Current approaches to study the role of large genomic regions rely on the simultaneous introduction of two sequence-specific DSBs, which when occur in the same DNA molecule will lead to the excision of the intervening region and eliminate the intended sequence. However, there is an inverse relation between the distance of the DSBs with the frequency of the deletion in the population of targeted cells^13^. These engineered events become increasingly rare when trying to model megabase-sized deletions as the ones observed in cancer genomes. Similar limitations occur in studies aimed to dissect the function of large DNA regions in transcriptional regulation and/or genome organization.

### Development of the protocol

**Figure 1:**
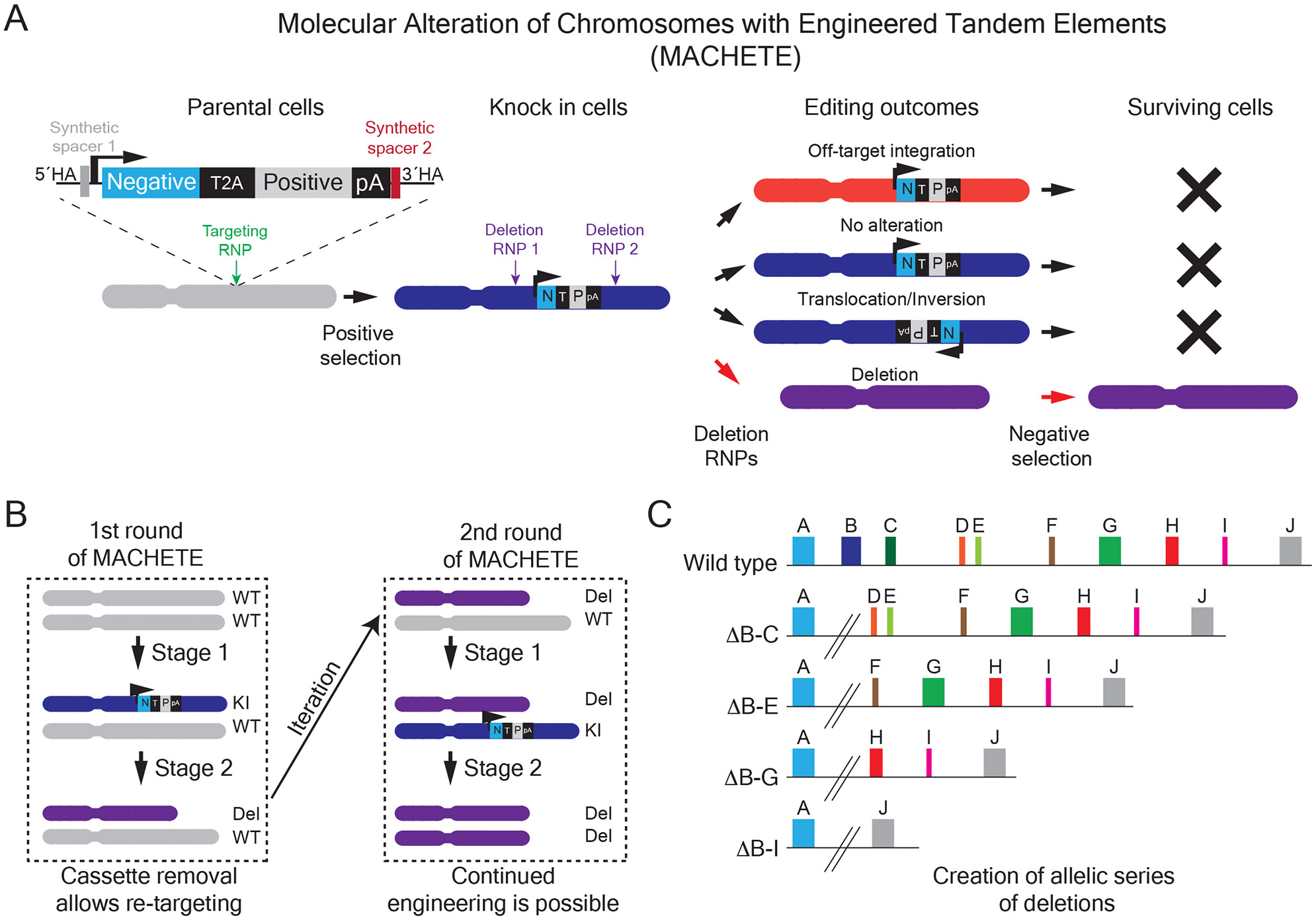
MACHETE allows engineering large genomic deletions. A. Schematic outline of the MACHETE protocol. A dual selection cassette is integrated via CRISPR HDR into the locus of interest. Cells with stable integration of the cassette are enriched via positive selection, ensuring the presence of the suicide cassette in the population. These knock-in cells are then used to engineer the intended deletions, which is followed by negative selection. Only those cells that excised the locus will survive the negative selection, greatly enriching for cells with deletions. The cassette excision occurs in a sequence-specific manner, thus eliminating cells with off-target integrations of the cassette. B. MACHETE can be iterated. Given that all exogenous elements are removed at the end of the procedure, deletions can be sequentially engineered either in the same locus or at a different one. C. MACHETE enables the creation of allelic series of deletions of a locus of interest. Using cells with a knock in of the dual selection cassette in the locus of interest, MACHETE allows creating allelic series of deletions of different size (e.g. ΔB-C, loss of the region spanning genes B and C; ΔB-E, loss of the region spanning genes B and E; etc).

To circumvent the inherent inefficiency of retrieving cells with large deletions, we devised an approach termed Molecular Alteration of Chromosomes with Engineered Tandem Elements (MACHETE)^14^. The basic premise of MACHETE is that the removal of a region of interest will give cells a selective growth advantage independently of the elements within the targeted locus. To achieve this, we use CRISPR-Cas9 mediated homology directed repair (HDR) to introduce an inducible suicide cassette into the locus of interest, which is positively selected by a linked antibiotic resistance or fluorescence gene (Figure 1). The integration of this tandem inducible suicide and selection cassette creates a molecular handle to enrich or deplete targeted cells as needed. Upon CRISPR-mediated introduction of cassette-flanking DSBs, only cells that eliminate the locus have a route to escape negative selection, greatly enriching for cells with the deletion of interest (Figure 1A). There are several unique features of MACHETE: first, since the cassette and locus are deleted in a sequence-specific manner via CRISPR-Cas9, our method eliminates cells containing off-target integrations of the cassette. Second, at the end of the protocol all exogenous elements are removed from the cell, allowing for iterative engineering of deletions (Figure 1B). Third, the high level of enrichment allows engineering of deletions of vastly different sizes across a locus, allowing refined mapping of megabase-sized regions (Figure 1C).

### Applications of the method

Genetic approaches have enabled us to identify the role of specific loci, which has been particularly focused on the biology of coding genes. MACHETE adds another tool to the genetics toolkit to facilitate understanding the biology of larger genomic regions.

**Figure 2:**
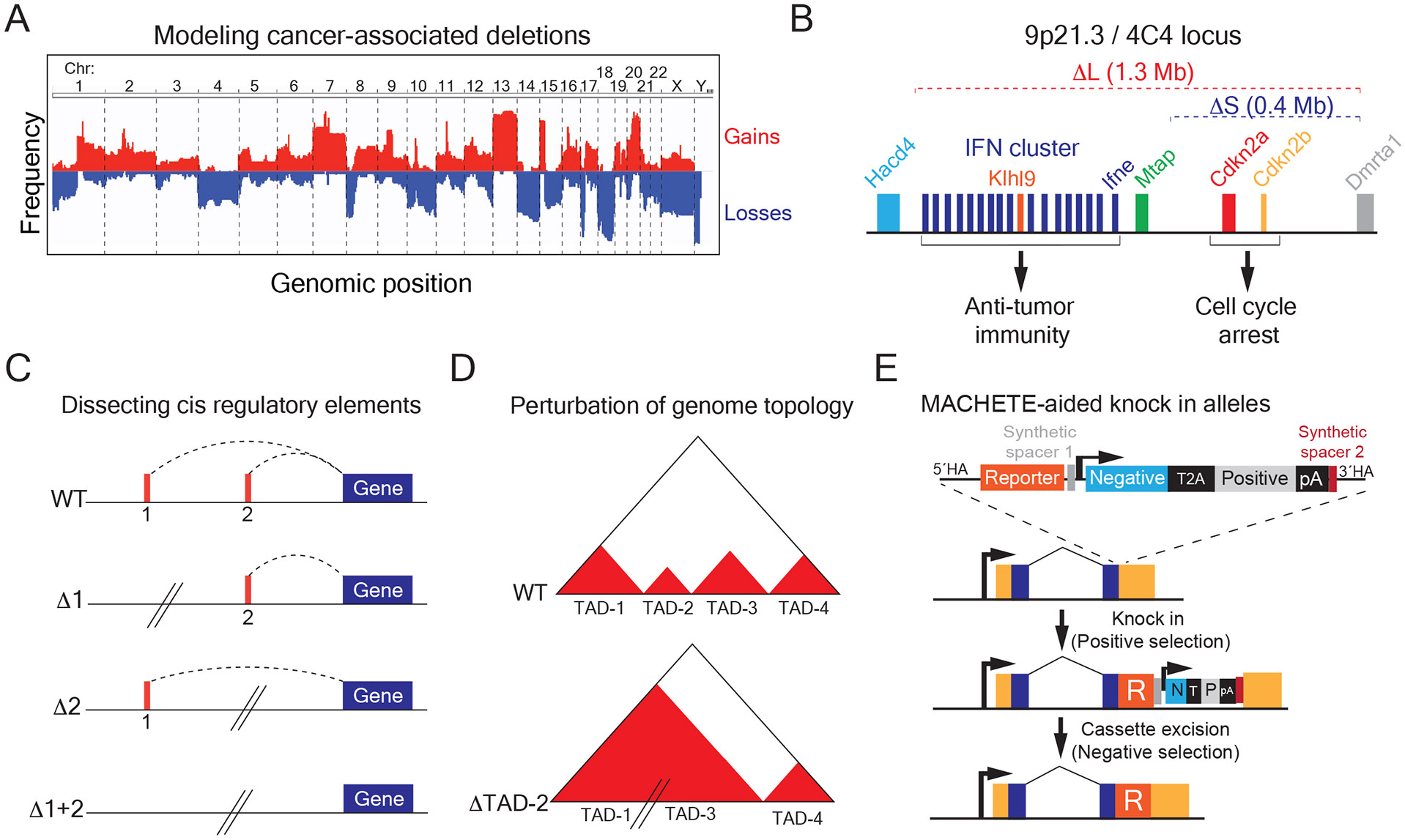
Applications of MACHETE. A. Schematic of Copy Number Alteration frequency in tumors, which guides the selection of target regions to engineer in cellular models via MACHETE. B. Chromosome 9p21.3 (syntenic to mouse 4C4) coordinates cell intrinsic and extrinsic tumor suppression. MACHETE engineering of the two most common configurations of 9p21.3 loss (ΔL and ΔS) identified the contribution of tumor-derived type I interferons in promoting immune surveillance and metastasis suppression. C. MACHETE can dissect the contribution of large cis regulatory elements to gene regulation. D. Potential application of MACHETE to elucidate genome structure/architecture via the functional interrogation of topological associated domains (TADs). E. MACHETE-aided integration of reporters into a locus of interest. Using the strong positive/negative selection and CRISPR-mediated excision of the MACHETE cassette can aid in the integration of reporters (e.g. fluorescent proteins, recombinases, etc) to loci that are poorly expressed.

The initial application of MACHETE is centered on the functional characterization of large deletions observed in cancer genomes (Figure 2A). Given their pervasiveness and potential clinical relevance, we decided to tackle this understudied aspect of cancer genetics. Our initial focus centered on understanding the loss of chromosome 9p21.3, which is the most frequent homozygous deletion (affecting 15% of all tumors) and portends poor prognosis in the cancers that have this genetic event^15^. The biological consequences of 9p21.3 deletions had been ascribed to the loss of the tumor suppressor genes *CDKN2A* (encoding p14^ARF^ and p16^INK4A^) and *CDKN2B* (encoding p15^INK4B^), whose protein products activate the RB and p53 pathways^16^. Thus, the classic view is that loss of 9p21.3 provides a strong proliferative advantage to cancer cells, which was validated in various cancer models. However, we and others observed that a subset of 9p21.3 deletions spanned an adjacent cluster of type I interferons (IFNs), which could account for some unexpected correlations of this deletion with alterations in tumor immunity. To dissect the role of tumor-intrinsic IFN loss we generated the two most common 9p21.3 deletions observed in patients: a 0.4 Mb allele eliminating *CDKN2A/B* and a 1.3 Mb allele eliminating both *CDKN2A/B* and the linked cluster of 17 type I IFNs. By applying MACHETE to mouse models of pancreas cancer and melanoma, we observed that IFN-deficient cancer cells were more metastatic and resistant to immune checkpoint blockade than IFN-proficient cells. These phenotypes were dependent on intact adaptive immunity, where tumor-derived IFNs activated antigen presenting cells and CD8 T cells. To identify whether one or more tumor-derived IFNs were involved in these effects, we created an allelic series of deletions that progressively eliminated the IFN cluster. An in vivo competition assay of these alleles identified that loss of interferon epsilon (*Ifne*) was a tumor-specific driver of these phenotypes in pancreas cancer^14^. This initial study illustrates the power of MACHETE in dissecting the complex effects that large genomic deletions can elicit on tumors (Figure 2B).

Beyond its use in dissecting the function of deletions in cancer, we envision MACHETE will be applicable to studying the contribution of any genomic region to multiple fields (e.g. transcriptional regulation, 3D genome structure, etc). The general design principle of the approach is applicable to any cellular system where negative selection can be implemented (Figure 2C-D).

Moreover, the MACHETE cassette can be leveraged as a tool to aid the delivery of other genetic elements into the genome. For instance, this is particularly relevant for introducing reporters into genes that are not expressed at baseline in cells of interest. The positive/negative selection then enables the excision of the MACHETE cassette to avoid the transcriptional interference of the strong exogenous promoter with the reporter-tagged endogenous gene (Figure 2E).

### Comparison with other methods

As outlined above, the most frequent methods to engineer deletions employ sequence-specific nucleases to create two DSBs simultaneously^13,17,11^, where CRISPR-Cas9 is the most prominent approach^18-20^. Optimizations of CRISPR-Cas9 have centered on increasing the editing efficiency by either enriching for cells that incorporate the components (e.g. via linked fluorescent or antibiotic resistance genes)^21^ or improving the rate at which DSBs are created (e.g. use of ribonucleoproteins, improved guide RNA scaffolds)^11^. However, these approaches do not circumvent the problem that most Cas9-expressing cells will not lead to the loss of the intervening region between two DSBs. To directly select for deletions, MACHETE relies on CRISPR-Cas9 to insert a selection cassette via HDR and subsequently create the intended DSBs, where negative selection robustly eliminates unedited cells.

Another approach to create deletions is the use of Cre recombinase coupled to flanking loxP sites^22^, and the major advantage of this strategy is the ability to create deletions in vivo. However, there are two limitations of the Cre-loxP approach: first, it requires the knock in of two loxP sites in cis. Second, loxP sites are at fixed positions, which only allows to create a single deletion per loxP pair. Overall, despite the potential advantages of Cre-loxP or other recombinases, the complexity of the targeting procedure and relative design rigidity has precluded its widespread implementation.

Finally, several approaches have been developed to model arm- and chromosome-level aneuploidy by using synthetic telomeres or disrupting chromosome segregation^23-25^. These approaches are very powerful tools to study aneuploidy yet have a relatively low frequency of on-target events and are not amenable to engineer interstitial deletions.

### Limitations

As MACHETE relies on CRISPR-Cas9, the limitations of this approach also apply to our method: most notably, these include challenges associated with off target effects and editing efficiency.

Off-target DSBs, and the generation of unintended structural variants, are known problems associated with CRISPR-Cas9^26,27^. To mitigate this problem, for each deletion we select two pairs of sgRNAs with the highest efficiency and lowest predicted off-target effects. Given that we target large genomic regions, there is ample room for identifying the best possible guide RNAs for any given locus of interest. The second issue, efficiency, is not directly addressed by our approach. However, since we rely on positive and negative selection for the distinct steps of MACHETE, even low efficiency sgRNAs will be effective in our system, albeit the number of cells surviving the selection may be lower than for high efficiency sgRNAs. Regardless, the combination of selection and the use of multiple independent guide RNAs circumvents most of the problems associated with CRISPR-Cas9.

Another limitation of MACHETE, and all other approaches to create deletions, is the limited multiplexing that can be implemented. Although negative selection enables retrieving rare cells with large deletions, in a pooled experiment smaller deletions would be the dominant outcome of the engineering, and larger deletions would be underrepresented in the initial pool. With these considerations, our experimental design of choice is to create distinct deletions independently, which if needed can then be pooled with equal representation for downstream applications.

Depending on the application, another limitation of MACHETE is that the default outcome is heterozygous deletions. Although in the context of cancer this is not an issue since most deletions are heterozygous, this may present a problem in applications where biallelic targeting is needed. To address this, we have showed that MACHETE can be iterated, allowing for the generation of homozygous deletions^14^. However, the size of homozygous deletions will be limited by the presence of essential genes/regions within the locus.

Finally, since MACHETE relies on strong negative selection to eliminate unedited cells, currently it cannot be used for somatic in vivo engineering of deletions, which would lead to organism failure if the selection cassette is present in all tissues.

### Experimental design

MACHETE is a two-stage procedure: first, an initial integration of the dual selection cassette into the locus of interest is achieved through CRISPR-Cas9 HDR and positive selection. Second, the introduction of sgRNAs is undertaken to eliminate the locus of interest followed by negative selection to enrich for cells bearing the desired deletion. Given the need for initial targeting and two rounds of selection, we recommend the application of MACHETE for deletions where the frequency of the on-target event is rare (e.g. deletions of over 1 Mb), smaller deletions where direct use of CRISPR-Cas9 has failed, or when heterozygous alterations are required.

The experimental design we favor involves the generation of at least one control allele (e.g. MACHETE cassette excision and/or a focal deletion) to compare with the experimental allele. Large deletions are engineered with two distinct sets of sgRNAs. This approach ensures that all cells have gone through a similar selection process and minimizes the possibility that phenotypic differences are driven by off-target DSBs or structural variants. Of note, in our experiments we worked with polyclonal populations due to the high efficiency of MACHETE in our systems, yet the protocol is compatible with the use of clonal populations if warranted.

**Figure 3:**
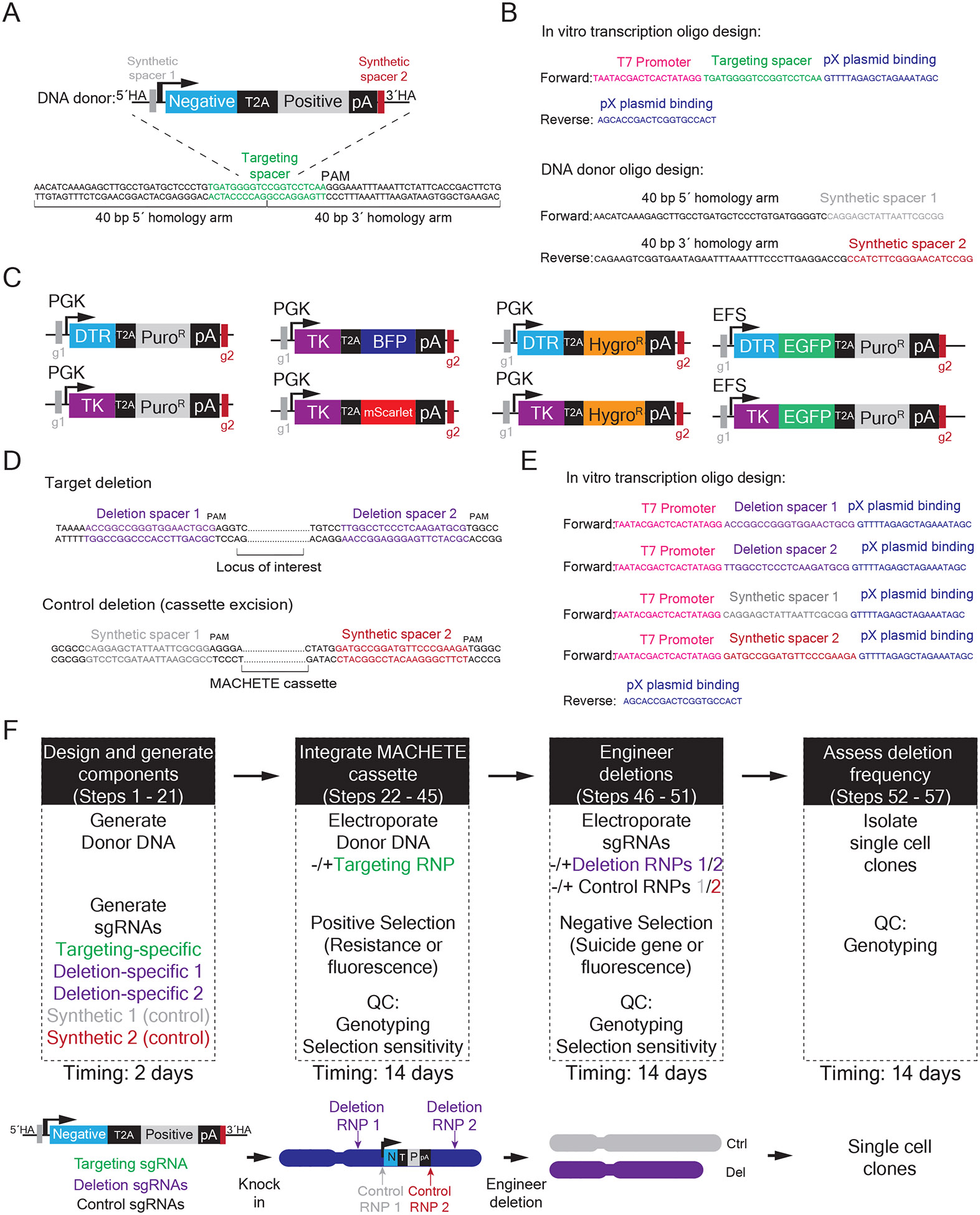
Design principles for the implementation of MACHETE. A. Schematic of a generic donor to be integrated into a region of interest. In green is highlighted the selected knock in spacer sequence, and the design of the 40 base pair homology arms is shown. B. Sequence of the oligonucleotides that are needed to make the targeting sgRNA and the PCR donor to the region highlighted in A. C. Available MACHETE cassettes (adapted from^14^). D. Schematic of the sequences that will define the intended deletion breakpoint (upper) and the control cassette excision (lower). In purple are highlighted the flanking spacer sequences, and in grey and dark red are highlighted the synthetic spacers to excise the MACHETE cassette. E. Sequence of the oligonucleotides that are needed to make the target and control deletion sgRNAs shown in D. F. Overview and schematic of the MACHETE protocol.

In Figure 3 we outline the experimental design and steps needed to implement MACHETE into any cellular system of interest.

#### Design and production of sgRNAs. Steps 1 – 14.

To engineer large deletions, we design guides targeting intergenic regions to minimize the potential effect on adjacent coding genes. We use the Guidescan database^28,29^, whose genome wide predictions and simple format allow the prioritization of the most specific guides with minimal off-target effects. One sgRNA is used for the CRISPR-Cas9 mediated cassette knock in (Figure 3A-B), and two pairs of guide RNAs are designed to target the flanking regions (Figure 3D-E). For MACHETE to be effective, the knock in sgRNA must be located between the flanking sgRNAs, anywhere within the intervening region between the intended DSBs . We favor knocking in the cassette closest to a specific element/gene of interest, which will enable focused mapping of that sub-region and comparison to larger deletions. Given that broken end resection of DSBs can expand in the order of several kilobases, if possible, we avoid placing the cassette too close to the intended breakpoint.

Guide RNAs are made via in vitro transcription of a PCR product bearing the T7 promoter, target spacer, and tracr sequence as previously described^30^. The in vitro transcribed approach reduces costs, yet chemically modified commercial guide RNAs can also be used.

#### Generation of PCR donor template. Steps 15 – 21.

The initial step of MACHETE relies on the integration of the dual selection cassette through CRISPR-Cas9 HDR. This requires a DNA repair template around the site of the designed DSB, to which 40 bp homology arms are introduced via PCR. This PCR based strategy precludes the need for cloning homology arms into the vector, reducing experimental time and increasing the flexibility of the approach. This size of homology arms was chosen because this length is sufficient to facilitate knock ins^31^ and allows use of shorter oligonucelotides to reduce costs. The template for the donor PCR can be any plasmid containing positive/negative selection elements. To aid in the implementation of MACHETE, we deposited in Addgene a collection of plasmids with various tandem positive and negative selection markers for use in mouse or human cells. All plasmids have shared flanking regions, which allow the amplification of any of the cassettes of interest with the same plasmid-binding oligo sequences (Figure 3C). These shared regions also contain distinct synthetic sgRNA binding sites absent in the mouse and human genomes, and which can be used to specifically excise the cassette. Thus, primers are designed as 60 mers comprised of the 40 bp of homology arm and 20 bp of plasmid binding sequence. To ensure the Cas9 RNPs do not cut the donor PCR, we design primers so that the end of the 5´homology arm is the 1-10 bp of the knock in sgRNA spacer sequence; conversely, the beginning of the 3´homology arm is the remaning 11-20 bp of the knock in sgRNA spacer sequence. We optimize the amplification conditions to ensure only the expected band is produced, which enables the donor fragment to be isolated at a high concentration via column-based PCR purification. In general, we avoid gel purification due to the reduced yield and low DNA quality associated with these approaches.

#### Targeting of the DNA donor into the locus of interest (Electroporation, Positive Selection, Drug sensitivity, QC). Steps 22 – 45.

To introduce the cassette into the locus of interest, we use CRISPR-Cas9 ribonucleoproteins (RNPs) and the donor DNA with 40 bp homology arms. Electroporation is used to introduce the RNP and DNA into target cells, although other delivery methods can be used. Transfected cells are left to recover for 48 hours and are then selected with the antibiotic or fluorescent marker of choice. As control, we include untransfected cells and cells receiving the donor DNA alone. After selection, surviving cells are expanded, tested for sensitivity to the negative selection, and genotyped for the presence of the on-target integration of the cassette.

#### Generating the deletion (Electroporation, Negative Selection, Sensitivity, QC). Steps 46 – 57.

To generate the deletion of interest, RNPs with two flanking guides are electroporated into cells containing the cassette. As for the initial step, if needed the use of fluorescently-labeled Cas9 can be used to estimate delivery efficiency and isolate cells that received the RNPs. Cells are left to recover for 48 hours after electroporation and are then cultured with negative selection. As controls, we use untransfected cells, or cells transfected with RNPs that specifically excise the cassette with minimal disruption of the genome (e.g. via synthetic guides in the donor DNA or guides directly flanking the cassette in the genome). We incorporated synthetic sgRNAs (absent in the mouse or human genomes) flanking the positive/negative selection cassette in all MACHETE plasmids. This allows excising the cassette itself without affecting the rest of the cell’s genome, which can be optionally used as a control for the procedure. These populations are compared to the cells receiving the sgRNAs used to create the deletions of interest. The cells surviving the negative selection are expanded and tested for sensitivity/resistance to the selection agents. To assess if the deletion engineering was successful, genomic DNA is analyzed for the intended breakpoint and presence/absence of the selection cassette. Further characterization of the deletion event can be done via derivation of single cell clones to evaluate the frequency and zygosity of the event in the population. The cells with control or experimental deletions are then used for downstream applications to assess their biological effects, where the specific phenotypic assays will be tailored to the project of interest.

#### Recommended controls.

##### Quantification of background events:

MACHETE enables the enrichment of deletions that can be rare within a population of cells, so it is important to ensure that the surviving cells are true deletion events and not due to incomplete selection. For this, we always include control cells that are electroporated but that do not include Cas9 RNPs for both stages of MACHETE.

##### Minimizing off-target effects (OTEs):

Given the well documented effect of CRISPR-Cas9 in creating DSBs in unintended regions, we always include two independent sets of sgRNAs to engineer every deletion of interest. This minimizes the chance that any given phenotype is driven by off target effects of specific sgRNAs. If warranted, testing for off-target effects can be incorporated by assessing indels in the predicted sgRNA binding sites. However, given the ample space for sgRNA selection, all the guides we have used have no predicted off targets (with 2 or 3 mismatches). Importantly, we have not observed unintended large deletions by sparse whole genome sequencing. In our experience the combined use of independent sgRNA pairs with the underlying features of the method ensures that OTEs are negligible.

##### Cassette excision (Optional):

The current design of the MACHETE plasmids includes the presence of synthetic spacer sequences flanking the selection cassette. These spacers are not present in the mouse or human genomes and enable the seamless excision of the selection cassette to obtain control cells that went through the same selection stages as the cells with larger deletions.

## MATERIALS

### Biological materials

#### Cell lines:

we have implemented MACHETE across a range of human and mouse cell lines (i.e. HEK293 cells, NIH3T3 cells, mouse embryonic stem cells, pancreatic ductal epithelial cells, B16F10 melanoma cells). Cell lines will be project-specific and should be cultured following the manufacturer or ATCC guidelines.

#### CAUTION:

The cell lines used in your research should be regularly checked to ensure they are authentic and are not infected with mycoplasma.

### Reagents

#### Cell culture:

Cell culture media for mammalian cells used.

PBS 1X (Thermo Fisher, cat. no. 14200083)

Trypsin-EDTA 0.25% (Gibco, cat. No. 25200-056)

Puromycin 10 mg/mL (Invivogen, cat. no. ant-pr-1)

Hygromycin B 50 mg/mL (ThermoFisher, cat. no. 10687010)

Diphtheria toxin, unnicked, Corynebacterium diphtheriae (Sigma Aldrich, cat. no. 3223261MG). CAUTION. Toxic when inhaled or orally. Work under a hood wearing a lab coat and disposable gloves.

Ganciclovir (Sigma Aldrich, cat. no. 345700-50MG). CAUTION. Is a potential cancer hazard.

Work wearing a lab coat and disposable gloves.

#### Molecular Biology reagents:

Locus specific 60-mer forward oligo: (5´- N_40bp_ TGCAGGAGCTATTAATTCGC-3´), where N_40bp_ refers to the 5´ homology arm, the remaining 20 bp bind to the 5´ sequence flanking the selection cassette in the collection of MACHETE plasmids (IDT)

Locus specific 60-mer reverse oligo: (5´- N_40_ GACCATCTTCGGGAACATCC-3´), where N_40bp_ refers to the 3´ homology arm, the remaining 20 bp bind to the 3´ sequence flanking the selection cassette in the collection of MACHETE plasmids (IDT)

sgRNA specific 59-mer forward oligo: (5´-TAATACGACTCACTATAGG N_20bp_ GTTTTAGAGCTAGAAATAGC-3’), where N_20bp_ refers to the sgRNA/spacer sequence (IDT)

sgRNA universal reverse oligo: (5´-AGCACCGACTCGGTGCCACT-3´) (IDT)

pX330 plasmid (Addgene, plasmid no. 58778)

Plasmids containing dual selection cassettes (e.g. MACHETE plasmid collection deposited in Addgene, plasmids no. 195282, 195283, 195284, 195285, 195286, 195288, 195289, 195290).

Q5 High-Fidelity 2X Master Mix (NEB, cat. no. M049L)

UltraPure DNAse/RNAse-Free Distilled Water (Thermo Fisher, cat. no. 10977035)

Agarose (Fisher Scientific, cat. no. BP160-500)

UltraPure DNA Typing Grade^™^ 50X TAE Buffer (Thermo Fisher, cat. no. 24710030)

SYBR Safe DNA Gel Stain (Thermo Fisher, cat. no. S33102). CAUTION. Is a potential cancer hazard. Work wearing a lab coat and disposable gloves.

QIAquick PCR purification kit (Qiagen, cat. no. 28104)

HiScribe T7 High Yield RNA Synthesis Kit (NEB, cat. no. E2040S)

RNA Clean & Concentrator (Zymo Research, cat. no. R1017)

TrueCut Cas9 Protein v2 (Thermo Fisher, cat. no. A36498).

Optional: Cas9-EGFP (IDT, cat. no. 10008100) or Cy3-Cas9 (PNA Bio, cat. no. CP06)

Neon NxT Electroporation System 10 μL kit (Thermo Fisher, cat. no. N1096)

Crystal violet solution (Sigma Aldrich, cat. no. V5265-500ML). CAUTION. Contains formaldehyde, methanol, and is toxic. Work under a chemical hood while wearing a lab coat and disposable gloves.

GoTaq Green Master Mix (Promega, cat. no. M7122)

DNA ladder 1 kb (Thermo Fisher, cat. no. SM0311)

DNA ladder 100 bp (Thermo Fisher, cat. no. 15628019)

6X gel Orange G loading buffer (Thermo Fisher, cat. no. R0631)

DNeasy Blood and Tissue kit (Qiagen, cat. no. 69504)

HCl 1M (Sigma, cat. no. 7647-01-0) CAUTION. HCl is corrosive. Wear gloves and a lab coat when handling.

NaOH 1M (Sigma, cat. no. 1310-73-2) CAUTION. NaOH is corrosive. Wear gloves and a lab coat when handling.

Trypan blue 0.4% (Gibco, cat. no. 15250061).

### Equipment

#### Cell culture:

Cell culture incubator, 37 C, 5% CO2

Cell culture laminar flow hood

100 cm^2^ tissue culture dish (Corning, cat. no. CLS430167)

6-well tissue culture plates (Corning, cat. no. CLS3506)

24-well tissue culture plates (Corning, cat. no. CLS3527)

96-well tissue culture plates (Corning, cat. no. CLS3527)

Falcon conical tubes 50 mL (Thermo Fisher, cat. no. 352070)

Falcon conical tubes 15 mL (Thermo Fisher, cat. no. 352096)

Neon Transfection System (Thermo Fisher, cat. no. NEON1SK)

TC20 automated cell counter (BioRad, cat. no. 1450102)

Cell counter slides (BioRad, cat. no. 1450003)

Benchtop Centrifuge 5810R, swinging bucket (Eppendorf)

Glass Pasteur Pipettes (Thermo Fisher, cat. no. 11775098; sterilized)

Water bath, 37 C

#### Molecular biology:

0.2 mL PCR tube strips with caps (Axygen, cat. no. 10810721)

96-well PCR plates (Brand, cat. no. 10279880)

Adhesive PCR plate-sealing sheets (Brand, cat. no. 781390)

1.5 mL PCR tubes (Eppendorf, cat. no. 15178344)

Benchtop centrifuge 5425R, fixed rotor (Eppendorf)

T100 PCR Thermal cycler (BioRad)

Agarose Gel Electrophoresis System (BioRad)

Chemidoc Gel Imaging system (BioRad)

Nanodrop 8000 spectrophotometer (Thermo Fisher)

Plate rocker (Thermo Fisher)

### Reagent setup

#### Oligos

Resuspend oligos to 100 μM in nuclease-free water. Make a 10 μM working solution of each oligo to use in the PCR reactions. Oligos can be stored at −20 °C for over 12 months.

#### TAE buffer

To make 10 L of TAE 1X, dissolve 200 mL of 50X TAE in deionized water. TAE buffer is stored at room temperature (24 °C) and should be used up to 6 months after preparation.

#### Diphtheria toxin

Resuspend diphtheria toxin in 1 mL of PBS 1X to create a stock solution of 1 mg/mL, and store as 50 μL aliquots at −80 °C. Prepare a 50 μg/mL working solution, store at −80 °C and avoid repeated freeze/thaw cycles. Diphtheria toxin aliquots can be stored at −80 °C for at least 12 months.

#### Ganciclovir

Resuspend ganciclovir in distilled water and adjust solution to pH = 12 with NaOH 1M. Once ganciclovir is in solution, adjust to pH = 11 with HCl 1M to create a working solution of 10 mg/mL. Filter through 0.22 μm to sterilize the solution and make 1 mL aliquots. Store at −20 °C, and once thawed do not freeze again. Thawed solution can be kept at 4 °C for up to 4 weeks.

### Equipment setup

#### Neon Transfection System

Set the specific program for the cells of interest Insert the pipette holder containing 3 mL of buffer E to receive the pipette with the electrode. Same holder can be used for all conditions of a given cell line, yet we swap the holder between different parental cell lines. CRITICAL: if conditions are not established for the cell line of interest, follow the optimization protocol to ensure efficient delivery of components.

## PROCEDURE

### Single guide RNA design.

Timing: 20 min per locus.

1. Identify the genomic coordinates of the locus of interest using a browser of choice (e.g. UCSC genome browser, ENSEMBL, NCBI).
2. Use GuideScan (www.guidescan.com) to identify an sgRNA to use for the cassette knock in and two pairs of flanking sgRNAs targeting the deletion breakpoints. sgRNAs should be prioritized for those with predicted lowest off-target events and highest efficiency. CRITICAL STEP: correct guide design is fundamental for the application of MACHETE.
  Troubleshooting.
3. Copy the sgRNA sequence (remember to exclude the NGG PAM sequence) and insert between the T7 promoter sequence and pX330 binding site to create a 59-mer oligo sequence. Repeat for all required sgRNAs. The primer sequence should be: TAATACGACTCACTATAGG N_20bp_ GTTTTAGAGCTAGAAATAGC, where N_20bp_ refers to the sgRNA spacer sequence.
4. Order the universal reverse primer (5´- AGCACCGACTCGGTGCCACT-3´) and the locus-specific 59-mer oligos in individual tubes at a 25-nmol scale (desalted).

### Single guide RNA preparation.

Timing: 2 days.

5. Setup PCR reactions to create DNA templates for the in vitro transcription. This protocol describes 30 μL per reaction, yet these can be scaled up to 100 μL per sgRNA for increased yield.

**Table:** 

Component	Volume (μL)	Final concentration
2X Q5 Master Mix	15	1X
10 μM locus-specific oligo	1.5	0.5 μM
10 μM universal oligo	1.5	0.5 μM
pX330 (10 ng / μL)	1.0	0.33 ng / μL
Nuclease free water	11	-
6. Perform a PCR amplification in a thermal cycler with the following settings:

**Table:** 

Cycle Number	Denature	Anneal	Extend	Final
1	98 °C, 2 min			
2-34	98 °C, 10 s	58 °C, 10 s	72 °C, 20 s	
35			72 °C, 5 min	
36				16 °C, hold
7. Use 5 μL of each PCR reaction, add 1 uL of 6X loading buffer, and run in a 2% agarose gel at 130V for 30 minutes to ensure the presence of a single 110 bp band.
8. Purify the remainder of the PCR product using the Qiagen PCR purification kit, following manufacturer’s instructions. Elute in 20 μL of TE buffer 1X to increase the DNA concentration.
9. Measure concentration with Nanodrop. Store at −20 °C until needed.
  Troubleshooting.
  Pause Point: PCR products can be stored at −20 °C for at least 12 months.
10. Perform in vitro transcription of PCR products from Step 9 using the HiScribe T7 High Yield RNA Synthesis Kit, as follows (20 μL reaction per guide):

**Table:** 

Component	Volume (μL)	Final Concentration
10X reaction buffer	2	1X
ATP 100 mM	2	10 mM
CTP 100 mM	2	10 mM
TTP 100 mM	2	10 mM
UTP 100 mM	2	10 mM
T7 RNA pol Mix	2	-
DNA (500 ng) from Step 9	2	25 ng/ μL
Nuclease-free water	6	-
11. Incubate the in vitro transcription reaction at 37 °C for 4-16 hours.
12. Dilute 1 μL of the in vitro transcription reaction with 4 μL of nuclease-free water, add 1 μL of 6X loading buffer, and run in a 2% agarose gel at 130 V for 35 minutes. A band of about 100 bp is expected.
  Troubleshooting.
13. Purify the transcribed sgRNAs with the Zymo RNA Clean/Concentrator kit, following manufacturer’s instructions. Elute the sgRNAs in 20 μL of H2O to increase concentration.
14. Measure the sgRNA concentration with a Nanodrop. Aliquot and store at −80 °C. Avoid freeze/thaw cycles. CRITICAL STEP: high concentration sgRNAs are needed to minimize volume and avoid disruption of the electroporation conditions.
  Troubleshooting.
  Pause Point: sgRNAs can be stored at −80 °C for at least 12 months.

### HDR donor design and preparation.

Timing: 2 days.

15. Identify the homology arms that will be used for HDR. The approach we prefer to use is to select 30 bp upstream and 30 bp downstream of the selected sgRNA (for a total sequence of 80 bp). The 5´ homology arm would correspond to the first 40 bp and the 3´homology arm would correspond to the second 40 bp. This design ensures the sgRNA will not cut the donor DNA, as the target site will be separated by the intervening selection cassette.
16. Design and order two 60-mer oligos: one forward oligo containing the 5´ homology arm and one reverse oligo containing the 3´ homology arm. Oligos are as follows: Forward oligo: 5´- N_5´HA_40bp_ TGCAGGAGCTATTAATTCGC-3´, where 5´HA_40bp refers to the 40 nucleotide 5´ homology arm identified in step 16.
  Reverse oligo: 5´- N_3´HA_40bp_ GACCATCTTCGGGAACATCC-3´, where 3´HA_40bp refers to the 40 nucleotide 3´ homology arm identified in step 16.
17. Setup PCR reactions (30 μL per reaction, 4 reactions per locus to increase yield) to create DNA donor for HDR:

**Table:** 

Component	Volume (μL)	Final concentration
2X Q5 Master Mix	15	1X
10 μM locus-specific oligo	1.5	0.5 μM
10 μM universal oligo	1.5	0.5 μM
Plasmid with positive/negative selection cassette (10 ng / μL)	1.0	0.33 ng / μL
Nuclease free water	11	-
18. Perform a PCR amplification in a thermal cycler with the following settings:

**Table:** 

Cycle Number	Denature	Anneal	Extend	Final
1	98 °C, 2 min			
2-34	98 °C, 30 s	58 °C, 30 s	72 °C, 2min 30s	
35			72 °C, 5 min	
36				16 °C, hold
19. Use 5 μL of each PCR reaction, add 1 uL of 6X loading buffer, and run in a 2% agarose gel at 130 V for 35 min to ensure the presence of a single 2-3kb band (depending on the selected cassette).
  Troubleshooting.
20. Purify the remainder of the PCR product using the Qiagen PCR purification kit, following manufacturer’s instructions. Elute in 20 μL of 1X TE buffer to increase the DNA concentration.
21. Measure DNA concentration using Nanodrop.
  CRITICAL STEP: Template concentration should be at least >250 ng/μL to ensure electroporation solution is not diluted.
  Troubleshooting.
  Pause Point: PCR products can be stored at −20 °C for at least 12 months.

### MACHETE cassette knock in and QC.

Timing: 1 day for electroporation, 7-14 days for selection, 3 days for QC.

22. Prepare tubes for each electroporation reaction. Mix 1 μg of recombinant Cas9 with 1 μg of sgRNA from Step 14 (knock in sgRNA) per reaction and incubate at room temperature for 15 min to make the RNPs. Samples to prepare are blank (no DNA / no Cas9), Cas9 RNP alone (recombinant Cas9 + KI sgRNA from step 14) and Cas9 RNP to be later mixed with donor DNA from step 21 (see step 27).
23. While the RNP is incubating, wash the cultured cells with PBS 1X, trypsinize, and proceed to count them.
24. Transfer the required number of cells (250,000 cells per condition) to a new 15 mL Falcon tube and dilute to 10 mL with PBS 1X.
25. Centrifuge the cells at 300 g for 3 min at 4 °C, discard supernatant, and resuspend with 10 mL of PBS 1X.
26. Centrifuge the cells at 300 g for 3 min at 4 °C, discard supernatant, and resuspend in Neon buffer R (10 μL of buffer / condition).
27. Add the donor DNA from step 21 to the tube with the corresponding RNP complex from Step 22.
28. Add 10 μL of cell suspension (250,000 cells) from Step 26 to each tube prepared in steps 22 or 27: blank (from step 22), Cas9 only (from step 22), and RNPs + donor mix (from step 27). Mix the suspensions by thorough pipetting.
29. Add 3 mL of buffer E to the Neon electroporation plastic holder and insert it into the Electroporator.
30. Take 10 μL of each cell suspension with a Neon fixed-volume pipette using the electrode containing tips.
  CRITICAL STEP: avoid introducing bubbles into the fixed volume pipette tip, which will disrupt the electroporation.
31. Insert the electrode tip into the Neon holder from Step 29 and electroporate the solution according to the cell line specific programs.
  CRITICAL STEP: Ensure you have identified the correct settings to deliver DNA and RNP to your cells. If not available, perform Neon optimization protocol to find the settings that maximize transfection while maintaining viability.
32. Transfer the electroporated cells to a well in a 6-well plate and let them recover for 48 hours. Repeat steps 30-32 for each tube prepared in step 28. Optional: if fluorescent Cas9 (e.g. Cas9-EGFP, Cy3-Cas9) was used, check cells with a fluorescence microscope or flow cytometer after 24 hours to confirm the presence of the RNPs.
33. Once electroporated cells reach 60-70% confluence, wash with 1X PBS, trypsinize, and passage them 1:1 into a new plate with media supplemented with either Puromycin (1 μg/mL) or hygromycin B (50 μg/mL).
  CRITICAL STEP: Optimize the antibiotic selection conditions for each cell line. We recommend a titration curve to identify the minimal dose that completely eliminates untargeted parental cells.
34. Grow the knock in cells in selection media to ensure cassette activity. To keep store stocks: Expand the population to three 10 cm culture plates. Once cells are 80-90% confluent, wash with 1X PBS, trypsinize, and take 90% of the cell suspension to centrifuge at 300 rpm for 5 min at 4 °C. Discard supernatant and resuspend the cell pellet of each 10 cm plate with 3 mL of cell culture media supplemented with 10% DMSO. Make 1 mL aliquots in cryotubes and freeze at −80 °C.
35. Keep the remaining 10% of cells from Step 34 in culture to genotype (to ensure the on-target integration, Steps 36 - 41) and assess drug sensitivity (functionality of the cassette, Step 42 - 45).
  Troubleshooting.
  Pause Point: cells can be stored at −80 °C for up to 3 months. For long term storage keep cells in liquid nitrogen.

#### Genotyping:

36. Design genotyping oligos for each knock in locus. Two PCRs are designed: an internal PCR for the cassette of interest, and a site-specific PCR by combining a genomic oligo upstream of the 5´homology arm and an internal oligo binding to the MACHETE cassette. In general, we design PCR products between 300-1000 bps.
37. Harvest 500,000 knock in and WT cells and centrifuge them at 300 g for 5 min at 4 °C. Extract genomic DNA from the pellet of parental and knock-in cells by using the Qiagen DNEasy Blood and Tissue kit, following manufacturer’s instructions.
38. Measure genomic DNA concentration using a Nanodrop spectrophotometer.
39. Setup PCR reaction using GreenTaq GO (20 μL / reaction) as follows. Include negative controls (e.g. blank, unrelated knock-in, and/or unrelated cassette):

**Table:** 

Component	Volume (μL)	Final concentration
2X Green Taq Go Mix	10	1X
10 μM locus- (on-target) or cassette-specific (internal) oligo	1.0	0.5 μM
10 μM cassette-specific oligo	1.0	0.5 μM
Genomic DNA (100 ng / μL)	1.0	5 ng / μL
Nuclease free water	7.0	-
40. Perform a PCR amplification in a thermal cycler with the following settings:

**Table:** 

Cycle Number	Denature	Anneal	Extend	Final
1	98 °C, 2 min			
2-34	98 °C, 30 s	58 °C, 30 s	72 °C, 30 s	
35			72 °C, 5 min	
36				16 °C, hold
41. Run samples in a 2% agarose gel at 130V for 35 min, and assess the presence of on-target and internal PCR products in the knock-in cells.
  Troubleshooting.

#### Knock-in cell drug sensitivity:

42. Seed 100,000 parental or knock-in cells in a 6 well plate. Leave cells untreated, add positive selection (puromycin 1 μg/mL or hygromycin B 50 μg/mL), or add negative selection (diphtheria toxin 50 ng/mL or ganciclovir 10 μg/mL). Culture cells for 48 to 72 hours.
  Alternatively, in the case of non-adherent cultures, measure relative cell survival by counting and using viability dyes (e.g. trypan blue): take a 10 μL aliquot of the cell suspension, add 10 μL of trypan blue, and mix by pipetting. Transfer 10 μL of this mix into a cell counter slide, and use automated counter to evaluate the fraction of living cells.
43. Remove media and stain cells with crystal violet solution for 20 min on a plate shaker.
44. Remove crystal violet solution, and wash plate by immersing in a container with running water 10 times.
45. Leave plates to dry and image in a ChemiDoc. Optional: plates can be imaged in a color scanner.
  Troubleshooting.

### Deletion engineering and QC.

Timing: 1 day for electroporation, 7-14 days for selection, 14 days for QC.

46. Prepare tubes for each electroporation reaction. Mix 2 μg of recombinant Cas9 with 1 μg of 5´ sgRNA and 1 μg of 3´gRNA (flanking sgRNAs prepared in Step 14) per reaction. Incubate this complex at room temperature for 15 min to make the RNPs. Prepare the following samples: Negative controls (e.g. blank, Cas9 alone), Cas9 RNPs to engineer intended deletions, and Cas9 RNPs to excise the cassette.
47. Follow Steps 23 to 32 for the electroporation of the tubes prepared in Step 46.
48. Trypsinize and passage the electroporated cells once they reach 60-70% confluence and plate into cell culture media supplemented with negative selection: diphtheria toxin for DTR constructs (50 ng/mL) or ganciclovir for TK constructs (10 μg/mL).
49. Expand the surviving cells under constant selection and freeze down several vials as in Step 34. Keep a fraction of cells in culture to test by genotyping (to ensure the presence of the breakpoints and loss of cassette) and drug sensitivity (functionality of the cassette).
  Troubleshooting.
  Pause Point: cells can be stored at −80 °C for up to 3 months. For long term storage keep cells in liquid nitrogen.

#### Genotyping:

50. Genotype cells (from Step 49) as described in Steps 36 – 41.

#### Deleted cell drug sensitivity:

51. Test sensitivity of cells (from Step 49) to selection agents as described in Steps 42 – 45.

### Assessment of deletion frequency by single cell clone genotyping: 10-14 days

52. Wash with 1X PBS, trypsinize, and centrifuge cells with the engineered deletions. Count cells and transfer 100-200 cells to a 15 mL conical tube with 10 mL of media. Plate 100 μL per well in each well of a 96-well plate.
53. 24 hours later, identify wells with individual cells under the miscroscope, and allow for clonal expansion. Change media every 72 hours.
  CRITICAL STEP: using the microscope ensure that single cells are observed in the wells that will be analyzed.
54. Wash cells with PBS 1X, trypsinize, and plate 10% of the cells in a well of a 24-well plate and expand in culture in the absence of any selection in standard media. Once cells reach 70-80% confluence proceed to freeze down vials as described in Step 34.
55. Transfer the remaining 90% of the cells of each clone (from Step 54) to a new Eppendorf tube with 500 μL of PBS 1X and centrifuge at 300 g at 4 C for 5 min.
56. Extract genomic DNA of the pellet from Step 55 with the DNEasy Tissue and Blood collection kit, following manufacturer’s instructions. Genotype cells following Steps 36 – 41.
57. Optional: if single cell clones are needed for downstream functional assays, identify wells with the expected deletions from Step 55, further expand, and freeze down a stock of cells as described in Step 34.
  Troubleshooting.
58. Use either polyclonal (from Step 49) or single cell clones (from Step 54) with the intended deletions for phenotypic characterization of cells. The specific downstream applications to assess the phenotypes of the deletions should be tailored to the project of interest and can include molecular, cellular, or in vivo assays.

## Troubleshooting

Troubleshooting advice can be found in Table 1.

## Timing

- Guide selection and design: 20 min per locus (Steps 1 – 4)
- Preparation of sgRNAs: 2 days (Steps 5 – 14)
- HDR donor design and preparation: 24 hours (Steps 15 – 21)
- MACHETE cassette knock in and QC: 10-14 days (Steps 22 – 45)
- Deletion engineering and QC: 10-14 days (Steps 46 – 51)
- Assessment of deletion frequency by single cell clone genotyping: 10-14 days (Steps 52 – 57)

## Anticipated results

**Figure 4:**
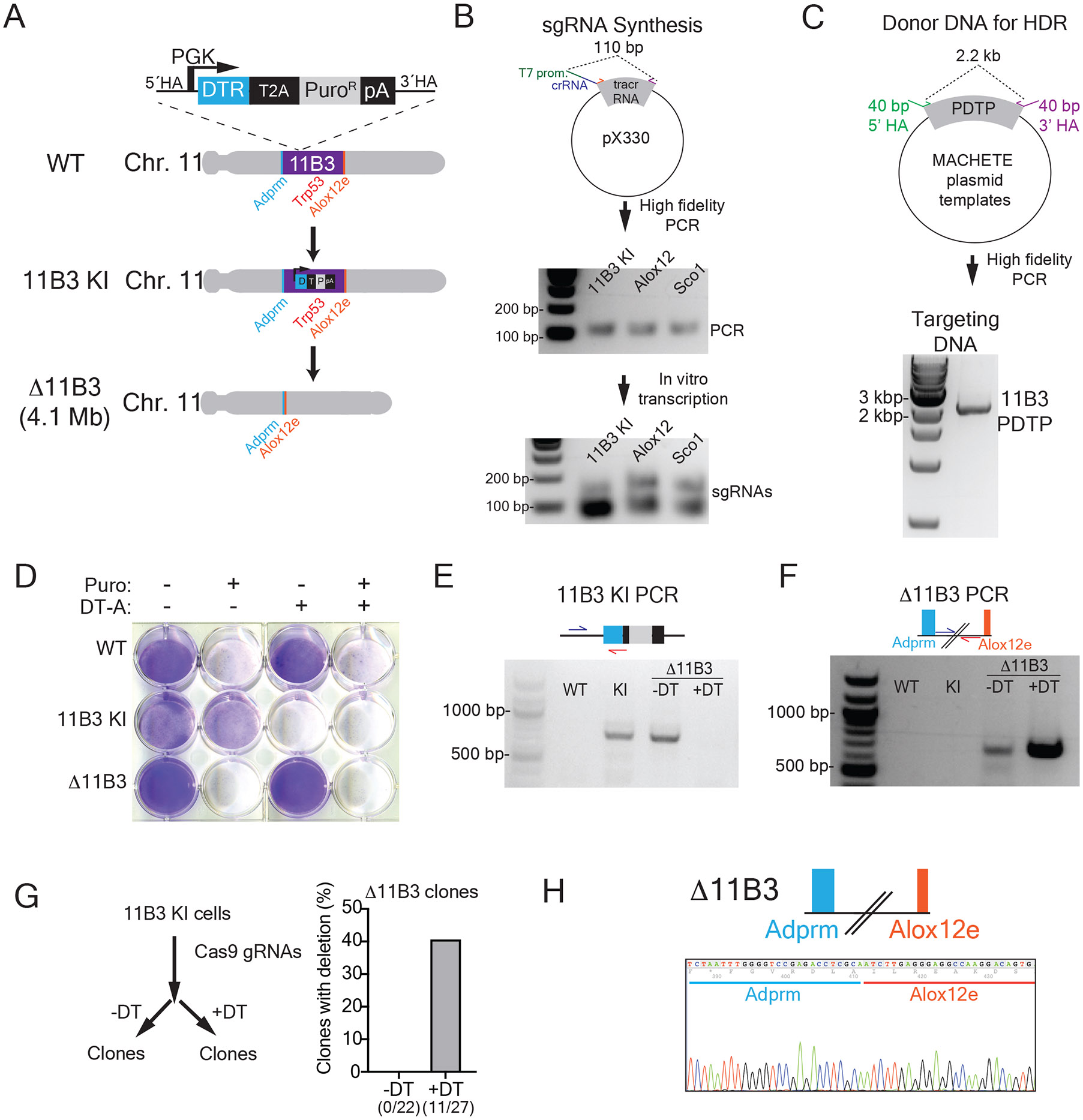
Expected Results (adapted with permission from^14^) A. Schematic representation of the design to create a 4 Mb deletion of the mouse 11B3 locus containing the tumor suppressor *Trp53* gene. B. Schematic of the procedure to synthesize knock in and deletion sgRNAs for the murine 11B3 locus. (Upper) Representative agarose gel of the PCR products used for in vitro transcription. (Lower) Representative agarose gel of the transcribed sgRNAs, which can appear as doublets in these non-denaturing agarose gels. C. Schematic and representative images of the procedure to create the DNA donor for CRISPR mediated HDR of a PGK DTR T2A Puro cassette into the 11B3 locus. Representative agarose gel of the DNA donor containing the MACHETE cassette and flanking homology arms used for HDR. D. Crystal violet staining of parental (WT), knock in, and deleted (Δ11B3) cells in the presence or absence of positive (Puromycin, 1 μg/mL) and negative (diphtheria toxin, 50 ng/mL) selection. E. PCR of the integration of the MACHETE cassette in the 11B3 locus. This was tested in the following cells: parental (WT), knock in (KI), unselected deleted (Δ11B3 – DTA), and selected deleted (Δ11B3 + DT-A). F. PCR of the engineered breakpoint of the 4.1 Mb Δ11B3 deletion. This was tested in the following cells: parental (WT), knock in (KI), unselected deleted (Δ11B3 – DT-A), and selected deleted (Δ11B3 + DT-A). G. Frequency of the intended D11B3 deletion in the population of unselected (− DT-A) and negatively-selected (+ DT-A) cells. H. Representative Sanger sequencing of the Δ11B3 breakpoint PCR confirming the intended junction in genomic DNA.

The PCR product containing the T7 promoter, spacer sequence, and tracr sequence is expected to be 110 bp (Figure 4B). The concentration of purified DNA for an effective transcription should range between 60-80 ng/μL. The in vitro transcribed sgRNA runs around the 100 bp marker, although in the non-denaturing agarose gel this may be seen as a doublet for some sequences. The in vitro transcription reaction yields around 100 μg of sgRNA, which is used at 1 μg/μL to reduce the volume in the electroporation reaction.

The PCR-based donor DNA will give a 2-3 kb band depending on the cassette of choice (Figure 4C). Gel purification should be avoided given the low yield of extraction. Electroporation conditions should be optimized for each cell line, although there are available conditions for most of the commonly used cell types. The first electroporation is done with 250,000 cells per condition, receiving 1 μg of Cas9, 1 μg of sgRNA, and 0.5-1.0 μg of donor DNA. We have observed little to no toxicity of the electroporation per se, and cells recover in less than 24 hours. For the positive selection step, negative controls (blank or no sgRNAs) will be eliminated, whereas as conditions receiving RNP + donor will generate cells bearing the selection marker. The speed at which selection occurs depends on the sensitivity of untargeted cells to the antibiotic and the doubling time of the resistant cells under selection. On average, it takes around 7 days to be able to expand sufficient cells for downstream targeting and quality control (via genotyping and drug sensitivity assays).

Having confirmed the presence of the on-target integration and sensitivity to negative selection (Figure 4D), these stable knock in cells can be stored long term and used for any deletions involving the targeted locus. Importantly, we keep cells under constant selection to ensure the cassette is retained. In our experience, the rate limiting step of the MACHETE protocol is the integration of the cassette given the low efficiency of HDR, yet the use of strong selection enables even rare events to be highly enriched.

With the established knock in lines, the procedure of deletion engineering is straightforward: electroporation of Cas9 RNPs with flanking guides, followed by negative selection. Cell populations are then characterized by genotyping the presence/absence of the knock in cassette and appearance of the intended breakpoints (Figure 4E-F). We have observed that the size of the deletion is inversely correlated with the number of cells surviving the negative selection. Our data shows that for deletions of more than 4 Mb, the use of MACHETE becomes enabling, where negative selection is required to detect the breakpoint event in clones (Figure 4G). Importantly, we have not detected off-target integrations in cells with deletions (tested via genotyping using internal cassette primers in cells that survived negative selection). As for positive selection, the speed of recovery of the cells will be related to the doubling time of the surviving population.

Our studies have relied on polyclonal populations, yet depending on the downstream application users may prefer to rely on single cell clones, whose characterization and expansion we have incorporated into the assessment of deletion frequency (Figure 4G-H).

## Supplementary Material

1

## Figures and Tables

**Table 1.** Troubleshooting.

Step	Issue	Possible reason	Solution
2	No guide with high efficiency or low off-target scores	Selected region contains repetitive elements	Use RepeatMasker to avoid repetitive elements. Change coordinates (1 kb upstream or downstream)
9	Low DNA concentration	Not enough template or inefficient PCR	Repeat PCR with more plasmid template or pool multiple reactions
12	Smear or no band in gel	sgRNA degradation	Repeat reaction and ensure nuclease-free conditions
14	Low sgRNA concentration	Poor in vitro transcription	Repeat reaction with more DNA template or pool multiple reactions
19	No amplification of donor DNA	Not sufficient template	Repeat reaction with more template
21	Low concentration of donor DNA	Low yield	Repeat by setting up multiple reactions per donor and pool for PCR purification
35	No cells survive positive selection	Low HDR efficiency	Repeat electroporation and scale up number of targeted cells. Alternatively, extend homology arms to 160 bp using an ultramer oligo for PCR
41	Undetectable on-target cassette integration	Genotyping PCR is not optimal (more common) or cassette is present only in off-target locus.	Re-design genotyping primers. Alternatively, change knock in locus.
45	Knock in cells are resistant to negative selection	Presence of untargeted cells due to incomplete positive selection	Increase concentration of positive selection. If needed, single cell clones can be derived.
49	No differential survival between negative control and targeted cells treated with negative selection	(i) Inefficient delivery of sgRNAs. (ii) sgRNAs do not efficiently create the intended DSBs.	(i)Repeat with increased concentration of sgRNAs. (ii) Design new sgRNAs targeting the intended locus.
57	Low frequency of cells with deletion	Negative selection not robust	Repeat deletion electroporation followed by more stringent negative selection. Cell number can be scaled accordingly to increase yield.

## Data Availability

All data related to this protocol is based on our previously published work^14^.

## Key points:

- MACHETE is a CRISPR-Cas9 based, two stage system to create megabase-sized genomic deletions: firstly, a bicistronic positive/negative selection cassette is inserted into the locus of interest, then single guide RNAs (sgRNAs) are added to engineer the intended deletion. Only cells that have lost the locus survive the negative selection, thus greatly enriching for cells with deletions.
- MACHETE circumvents the inefficiency of retrieving cells with large deletions that limits previous methods.

## Related links

References that have used this protocol: Barriga, F.M., Tsanov, K.M., Ho, YJ. et al. MACHETE identifies interferon-encompassing chromosome 9p21.3 deletions as mediators of immune evasion and metastasis. Nat Cancer 3, 1367–1385 (2022). https://doi.org/10.1038/s43018-022-00443-5
