# Supplementary material for: Engineering megabase-sized genomic deletions with MACHETE (Molecular Alteration of Chromosomes with Engineered Tandem Elements)
Source: Nat Protoc. Author manuscript; Available in PMC 2024 Dec 13. (PMC11642290; doi:10.1038/s41596-024-00953-9)

Source Data Figure 4

Figure 4A (top) gRNA PCR

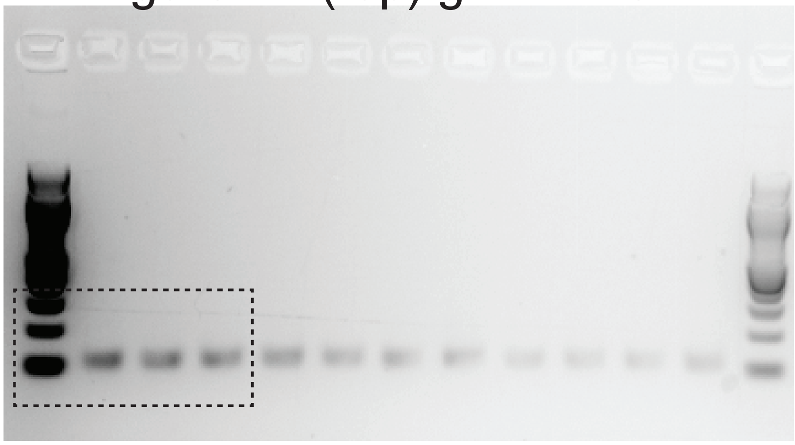

Figure 4C
Cassette PCR

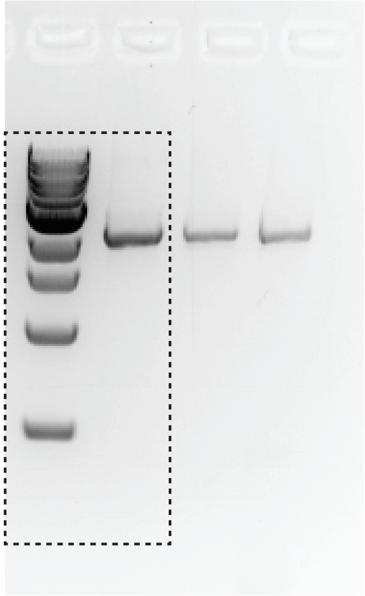

Figure 4B (bottom) IVT gRNAs

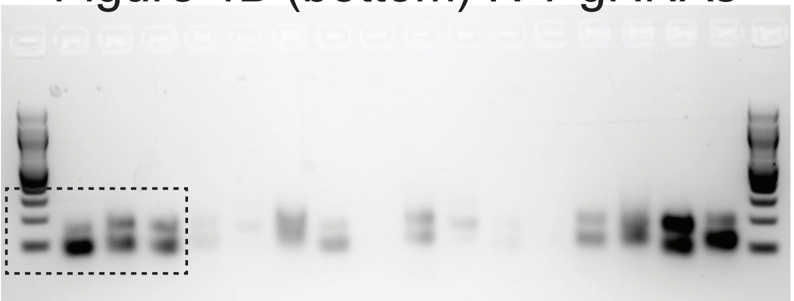

Figure 4E
KI PCR

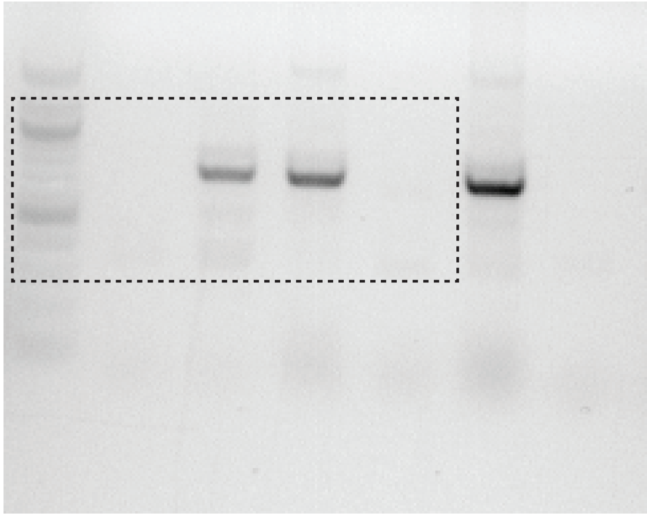

Figure 4F
Δ11B3 PCR

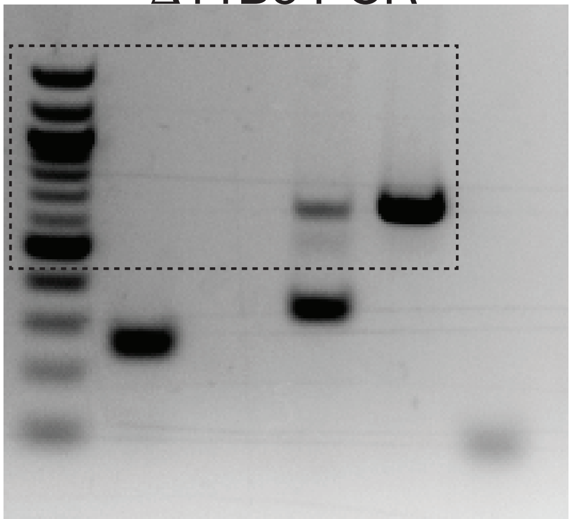

Supplement: 1 [file NIHMS2038981-supplement-1.pdf]
